# Research progress on hepatic machine perfusion

**DOI:** 10.7150/ijms.56139

**Published:** 2021-03-03

**Authors:** Junda Gao, Kang He, Qiang Xia, Jianjun Zhang

**Affiliations:** Department of Liver Surgery, Renji Hospital, School of Medicine, Shanghai Jiao Tong University, Shanghai, China.

**Keywords:** hepatic machine perfusion, hypothermic machine perfusion, subnormothermic machine perfusion, normothermic machine perfusion, liver transplantation, extended criteria donors

## Abstract

Nowadays, liver transplantation is the most effective treatment for end-stage liver disease. However, the increasing imbalance between growing demand for liver transplantation and the shortage of donor pool restricts the development of liver transplantation. How to expand the donor pool is a significant problem to be solved clinically. Many doctors have devoted themselves to marginal grafting, which introduces livers with barely passable quality but a high risk of transplant failure into the donor pool. However, existing common methods of preserving marginal grafts lead to both high risk of postoperative complications and high mortality. The application of machine perfusion allows surgeons to make marginal livers meet the standard criteria for transplant, which shows promising prospect in preserving and repairing donor livers and improving ischemia reperfusion injury. This review summarizes the progress of recent researches on hepatic machine perfusion.

## Introduction

At present, the shortage of donor livers remains very serious worldwide. Donor livers from dead Chinese citizens accounted for 83.55% of the total number of livers for transplantation by 2015. But only about 0.2% people were willing to donate organs after death in China. (https://www.codac.org.cn) The percentage of European patients waiting for donor livers for less than 3 months has fallen from 90% in the 1980s to slightly over 50% since 2000 1. In 2018, only 46.5% of American patients could get liver transplantation within one year. Each year, thousands of patients died of disease aggravation due to lack of qualified liver transplantation 2. In this regard, the discrepancy between donor organ demand and supply is increasing and patients are suffering from longer waiting list. Expanding the donor pool and using all available organs is an imperative need. Several methods, such as living donation, split-liver and marginal grafts, have been used to expand the donor pool. The most promising one is marginal grafts, which come from extended criteria donors (ECD) and donation after circulatory death (DCD) rather than standard criteria donors (SCD). However, the use of ECD or DCD often leads to adverse outcomes and increased risk of infection (Table 1) because they are more sensitive to ischemia-reperfusion injury (IRI) 3. Therefore, improving the quality of marginal grafts will improve the prognosis of patients to some extent.

Nowadays, static cold storage (SCS) is widely used to preserve donor livers. SCS slows down metabolism and reduces oxygen consumption by lowering the temperature of the donor liver, which is able to avoid rapid functional damage in a longer time of ischemia. However, preserving ECD or DCD by SCS is unsatisfactory 4. As an alternative method, machine perfusion may have more advantages. Machine perfusion can not only provide oxygen and nutrients for the liver, but also remove metabolic waste and reduce the damage to hepatic metabolism caused by warm ischemia or hypothermia. In addition, machine perfusion can detect the function of grafts *in vitro* before transplantation 5,6 and administer drugs for therapeutic intervention, which cannot be achieved by SCS. Lots of livers that could have been transplanted were abandoned due to the lack of objective and effective prediction and evaluation of the marginal function 7. And transplantation can also be avoided for the livers with irreversibly loss of function 8. Machine perfusion may change this predicament. A clinical randomized controlled trial by Nasralla et al. 9 showed that machine perfusion does contribute to improving utilization and prolong the lifespan of grafts. Under this background, machine perfusion is considered to be one of the great advances in the field of transplantation in decades 10. This review summarizes the progress of recent researches on hepatic machine perfusion.

## Mechanism of Machine Perfusion

**Figure 1:**
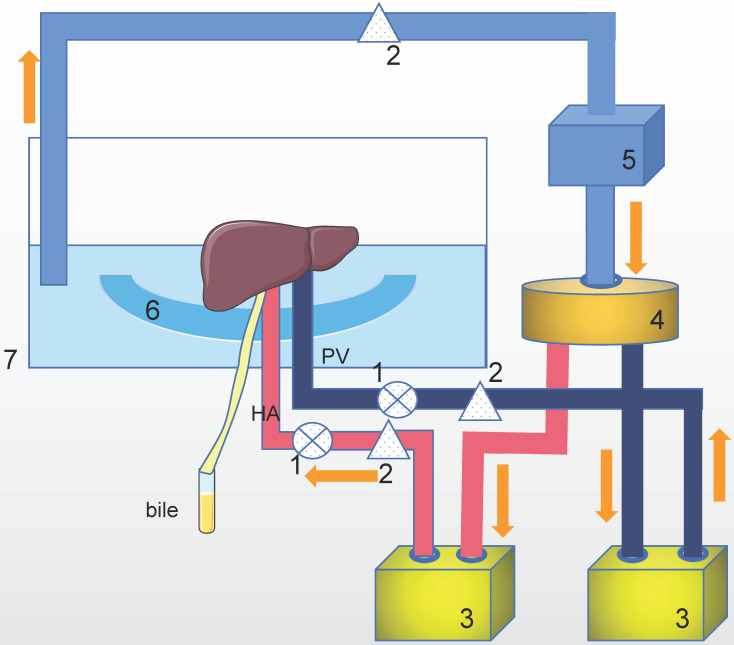
** The mechanism of machine perfusion.** This is only one example of a perfusion setup with a Y-configuration. There are some other setups with single (via the hepatic artery or portal vein) perfusion or two completely parallel circuits. Technical of oxygenation, pump and temperature control vary in different setups. HA: hepatic artery. PV: portal vein. The perfusion system includes: 1. Pressure senor, 2. Flow meter, 3. Pump, 4. Oxygenator/heat exchange, 5. Centrifugal pump, 6. Organ holder, 7. Organ chamber.

The purpose of machine perfusion is to maintain organ vitality, repair and pretreat the organs. During machine perfusion, the donor organ is usually connected to a pressure-controlled perfusion apparatus that continuously pumps the perfusate through the blood vessels of the organ (Figure 1).

There are three main methods for liver preservation by machine perfusion, from hypothermic (4-10 °C), subnormothermic (20-25 °C) to normothermic (35-37 °C) 18. No precise division of the temperature is defined. Another classification includes hypothermic (0-12 °C), subnormothermic (25-34 °C) and normothermic (35-38 °C) 19. Recently, a new concept of controlled oxygenated rewarming (COR) was put forward. The process of COR was gradually increase the graft temperature (up to 20 °C) with sufficient oxygenation of the liver tissue 20.

### Hypothermic Machine Perfusion (HMP)

Apart from achieving cooling effect same as SCS, HMP can also transport metabolic waste out of the donor liver and administer drugs. The easy transformation into SCS in condition of machine breakdown renders HMP a safe technique as well. A latest meta-analysis 21 showed that the incidence of post-transplantation one-year survival was increased with an OR of 2.19 (95% CI 1.14-4.20, *p*=0.02) in HMP preservation compared to SCS.

HMP can be performed through either portal vein (PV) or portal vein and hepatic artery (HA), also known as dual perfusion 22. In adult liver transplantation, the perfusion pressure of PV and HA are usually set to 3-5 mmHg and 20-30 mmHg, respectively 23. Werner et al. 24 reported the first case of pediatric DCD liver transplantation via HMP. Werner pointed out that the perfusion pressure of PV and HA in children should decrease proportionally in accordance with adults' mean arterial pressure. HMP has also been proved to have a good effect on preservation of split liver 25. Gillooly et al. 26 reported that adding short interfering RNA (siRNA) to the perfusate could inhibit chemically mediated liver failure in mice, suggesting that taking advantage of siRNA may bring a better outcome of liver transplantation.

Hypothermic oxygenated perfusion (HOPE), developed along with HMP, additionally provides oxygen to the donor liver at an approximate pressure of 60-80 kPa during perfusion 27. Studies have shown that HOPE performed shortly after graft extraction can reduce metabolic waste production, promote mitochondrial function recovery 28,29 and improve liver ATP level, thus ameliorating hepatic function 23 and reducing the incidence of post-transplantation intrahepatic biliary complications 30,31. Ravaioli 32 believes that HOPE should last at least an hour. The safety and efficacy of HOPE have also been confirmed. Rayar et al. 33 reported two cases of successful liver transplantation using HOPE to preserve ECD livers in their center. In one of the cases, the donor was over 80 years old, with steatosis greater more than 20% and cold ischemia time (CIT) greater than 10 hours. Inspiringly, the hepatic function recovered quickly after surgery, indicating that HOPE can improve the IRI and restore the function of the donor liver favorably. Even if choosing donor liver with advanced age means higher risk of post-transplant complication 34, the result of this research may still present elder donor liver as an option for surgeons. Schlegel et al. 35 compared the differences of the 5-year survival rate of DCD liver transplantation between HOPE and SCS. The results showed that the 5-year survival rate of the former was 94%, while that of the latter was only 78%. Despite higher risk of failure, the 5-year survival rate after DCD liver transplantation is similar to that of donation after brain death. HOPE can also extend the lifespan of donor livers 36,37, providing convenience for liver distribution.

### Subnormothermic Machine Perfusion (SNMP)

Subnormothermic machine perfusion attempts to maximize hepatic metabolism while minimizing reperfusion injury. With the temperature within subnormothermic range, the cell's demand for energy significantly declines while sufficient metabolism is maintained to monitor and restore liver function 38. The effect of SNMP on preserving DCD liver has been verified in pig liver transplantation 39. SNMP can reduce portal venous resistance and increase bile production compared with HMP, therefore better utilizing the DCD liver 40. Morito, N et al. 41 pointed out that SNMP consumes higher oxygen than HMP, which restores the function of the donor liver and reduces the incidence of endothelial cell injury, therefore making better use of DCD liver and expanding the donor pool. However, Kanazawa 42 pointed out that a noticeable delay exists between the beginning of machine perfusion and the full perfusion of the peripheral liver tissue. Short as it is, the delay exposes the insufficiently perfused areas directly to room temperature. In another word, SNMP is not as effective as HMP in protecting liver tissues with inadequate perfusion.

Ciria, R et al. 43 applied SNMP to extreme ECD, including long periods of cold and warm ischemia, and the restoration effect was inspiring. Although the post-transplantation prognosis is uncertain, lactic acid descending trend and bile output are prominent. At present, there is no agreed conclusion on whether it is beneficial to use oxygen carriers in SNMP. Shonaka, T 44,45 added hemoglobin-based oxygen vesicles (HbV) as oxygen carriers to UW solution in SNMP for porcine DCD liver, and proved that the reperfusion injury of the donor liver could be ameliorated. Karimian et al. 46 have shown that there are ATP accumulation, energy charge ratios and glutathione consumption in steatosis liver after SNMP, which may weaken the antioxidant capacity of grafts 47. Although there are no clinical reports of steatosis liver transplantation via SNMP, it is necessary to beware of the possibility of severe ischemia-reperfusion injury and a high incidence of EAD.

Huang, V et al. 48 reported that a split-liver perfusion model could be established by SNMP *in vitro*, which allows simultaneous perfusion of left and right lobes and one lobe to serve as control for the other. Similar to whole-liver perfusions, the arterial resistance and lactic acid levels of each lobe in the split-liver model decreased gradually during the whole perfusion, and there was no significant difference between the left and right lobes. This model avoids the problems caused by the heterogenous nature of discarded human liver that troubled previous researches, and is conducive to the development of preclinical studies. Obara, H et al. 49,50 suggested that installation of leukocyte filter or replacement of purified perfusate in SNMP could reduce hepatic artery pressure and release the levels of AST, LDH and ALP in porcine liver donated after cardiac death. This method is expected to further improve the ischemia-reperfusion injury in ECD. Tabka et al. 51 reported that the addition of angiotensin Ⅳ to the perfusate could improve the function of hepatic endothelial cells and reduce oxidative stress and cell injury. Future research may focus on optimizing the composition of perfusate to further improve the quality of graft and the prognosis of transplantation.

### Normothermic Machine Perfusion (NMP)

NMP provides oxygen and nutrients to the liver at 37 °C, keeping the liver in a complete functional state *in vitro* 52. The perfect mimicking of physiological condition enhances the metabolic activity of the donor liver and reduces the ischemic graft injury 53, allowing for more active repair and providing the opportunity for therapeutic intervention to a functioning organ before it is transplanted (Table 2). Therefore, NMP may be more helpful for the repair of ECD. One of the main advantages of NMP is that the functional status of grafts can be evaluated by measuring liver metabolic indicators such as bile production and liver enzymes before transplantation 54. The first case of ECD transplantation by NMP in Asia was reported recently 55. Multicenter randomized controlled trials have shown that NMP can reduce graft injury, prolong organ preservation time and improve organ utilization 9,56. Stephenson et al. 57 proposed the possibility of split-liver in NMP, and proved that the two parts of the split-liver could maintain function, meaning two cases of liver transplantation could be completed successfully.

Perfusate of NMP requires sufficient oxygen carriers to transport oxygen and maintain physiological osmotic pressure. At present, most perfusate use red blood cells as oxygen carriers. However, it has several disadvantages, including immune-mediated responses and hemolysis 58. Even so, the safety and efficacy of fresh frozen plasma in NMP were confirmed in controlled trials 59.

NMP is more technically challenging and expensive than SCS, especially during the preservation period, therefore more human resources are required. Selzner, M 60 believes that the maintenance process of the liver in NMP is extended 2 hours due to the processes of preparation, intubation, equipment connection, etc. Any failure in these techniques may have a disastrous impact, rendering the organ nonviable.

### Controlled Oxygenated Rewarming (COR)

The temperature of COR is similar to that of SNMP. Metabolism of the graft was decreased in SNMP, while increased in the process of COR. The first clinical trial of COR was carried out by Hoyer et al. 71 in 2016 with a short-term follow-up of 6 months. Six donor livers were stored by SCS and then performed COR with no prolonged cold ischemic time before transplantation in this study. Afterwards, they extended the clinical trial to 18 liver transplantation and assessed the long-term outcome of recipients 72. No significant decrease in postoperative outcome was observed. These studies demonstrated the feasibility and safety of COR in the clinic.

## Conclusions and Future Perspectives

There are many clinical trials on hepatic machine perfusion, but we are unable to completely replace SCS with machine perfusion due to economic factors and lack of strong evidence. At present, most experiments of machine perfusion are carried out on isolated liver models or transplantation studies in animal models, which cannot fully simulate the physiological environment and metabolism of the human body. More data from liver transplantation models are needed to verify some of the experimental results. Only in the case of ECD and DCD can the benefits of machine perfusion be predicted. However, what needs to be solved at present is that the indications for machine perfusion are not clear, which means there is no consensus on when should we consider machine perfusion 73. Moreover, there is no agreed idea on which machine perfusion is the best under different circumstances 74. Since organ transport takes a long time in most cases, the duration to achieve the most effective resuscitation of the donor liver has not been determined 42. There are also continuous studies on how to better evaluate graft function 75. With the development of machine perfusion research in transplant centers all over the world, each center has its own experience in choosing perfusion equipment, composition of perfusate, and perfusion time and evaluating graft function 76. Researches will focus on forming a consensus in order to facilitate its application.

In addition, combination of multiple perfusion methods may be promising in making better use of the advantages of different perfusion methods. For example 77-79, combining HOPE, controlled oxygenated rewarming (COR) and NMP can reduce oxidation-induced tissue damage and increase the energy storation of the liver. De Vries et al. 80 showed that human livers can be stored with the combination of supercooling below zero (-4 °C) and SNMP, effectively prolonging the duration of human liver preservation to 27 hours. Eshmuminov 81 and his team designed a modified perfusion machine, which can simulate the transport of nutrients and bile salt from the intestine to the liver through the portal vein. In order to keep the liver moving continuously, a device to mimic diaphragm oscillations was also integrated. At the same time, it can also control the hemodynamics of the liver and regulate the level of blood sugar through feedback. Six out of ten injured human livers that had been abandoned by all European centers were successfully preserved by this new system at 34 °C for a week and might meet the criteria for transplantation. With the ongoing development of machine perfusion, the definition of ECD will continue to be extended, but how to balance the pros and cons of the shortage of donor pool and marginal donors remains to be studied in the future.

## Figures and Tables

**Table 1:** Common complications of ECD and DCD

	Definition	Common complication after transplantation
ECD	No precise definition; Frequently cited characteristics are 11: Advanced age; macrovesicular steatosis; DCD; organ dysfunction. Cause of death: anoxia, cerebrovascular accident. Infectious disease: Hepatitis B, Hepatitis C, HIV. Extrahepatic malignancy. Cold ischemia time (CIT) greater than 12 hours.	Early allograft dysfunction (EAD); Biliary complication Recurrence of Hepatitis Poor prognosis (higher mortality) 12,13
DCD	Organs donated after cardiac death of the donors	EAD; Primary nonfunction (PNF); Biliary complication; Transplant failure 14-17

**Table 2:** Major categories of therapeutic intervention of *ex vivo* NMP in liver transplantation

Category	Agents	Advantages	Limitations/Further studies
Gene silencing with RNAi	SiRNA 26,61	Opened up new opportunities for specific therapeutic targeting of genes; More efficient delivery, lower doses and cost-saving; None/Fewer side effects to other organs; Organ-specific; Administration method clinically more applicable.	Risks of delivering the medication systemically; Chemical structural challenges related to uptake; Off-target effects; Application requires machine preservation expertise.
Cell therapy	Regulatory T-cell (Treg) 62	Alleviate or prevent graft-versus-host disease	Difficult to deliver therapeutic cells to target organ; Lack of standardisation of manufacturing processes; High cost
Extra-cellular vesicles	HLSC-EV 63	Reduce liver injury during hypoxic NMP; Mimic most of the cell effects (including apoptosis inhibition and mitogenic activity) by transferring proteins, mRNAs, and micro-RNAs Represent an innovative approach to recondition organs before transplant	Mechanism of HLSC-EV on reducing hypoxic injury unclear
Vasodilator agent	Prostaglandin E1 64,65	Improve extracellular stress associated with microcirculatory failure and hypoxia; Suppress the production of cytokines from macrophages and proinflammatory cytokine	Need more research on actual transplantation experiments using large animal models Frequency of biliary complications unclear.
Antibiotics/Antiviral agents	miravirsen 66	Make a liver resistant to reinfection; Lower risk for long-term complications.	Lack of suitable animal models; Need more longer-term follow-up studies.
Defatting cocktail	Combination of multiple defatting agents 67-69	Enhance lipid metabolism and mitochondrial functioning of hepatocyte; Improve organ functional recovery; Decrease vascular resistances; Anti-inflammation and reduce markers of hepatocellular injury Improve biliary function.	The effectiveness of the drugs after ischemic injury remain dispute; Lack of clinical markers for some defatting agents; Unknown livers after defatting cocktail suitable for transplantation.
Opioid agonist	Enkephalin 70	Protective against oxidative; Decrease the metabolic demand; May serve as therapeutic target for improved liver protection; Preserved the mitochondrial function of oxidatively stressed hepatocytes; Lengthen the preservation time of donor organs during normothermic perfusion.	Lack of strong clinical relevance; Need more research on test DADLE using alternative *in vitro* models of oxidative stress; Mechanism of DADLE on decreasing hepatocellular metabolism unclear.

siRNA: small interfering ribonucleic acid; HLSC-EV: Human liver stem cells extracellular vesicles; DADLE: delta opioid agonist [D-Ala2, D-Leu5] encephalin.

## References

[1] Adam R, Karam V, Cailliez V (2018). 2018 Annual Report of the European Liver Transplant Registry (ELTR) - 50-year evolution of liver transplantation. TRANSPL INT.

[2] Kwong A, Kim WR, Lake JR (2020). OPTN/SRTR 2018 Annual Data Report: Liver. AM J TRANSPLANT.

[3] Boecker J, Czigany Z, Bednarsch J (2019). Potential value and limitations of different clinical scoring systems in the assessment of short- and long-term outcome following orthotopic liver transplantation. PLOS ONE.

[4] Monbaliu D, Pirenne J, Talbot D (2012). Liver transplantation using Donation after Cardiac Death donors. J HEPATOL.

[5] Marecki H, Bozorgzadeh A, Porte RJ (2017). Liver ex situ machine perfusion preservation: A review of the methodology and results of large animal studies and clinical trials. Liver Transpl.

[6] Laing RW, Mergental H, Yap C (2017). Viability testing and transplantation of marginal livers (VITTAL) using normothermic machine perfusion: study protocol for an open-label, non-randomised, prospective, single-arm trial. BMJ OPEN.

[7] Collett D, Friend PJ, Watson CJE (2017). Factors Associated With Short- and Long-term Liver Graft Survival in the United Kingdom. TRANSPLANTATION.

[8] Mergental H, Stephenson BTF, Laing RW (2018). Development of Clinical Criteria for Functional Assessment to Predict Primary Nonfunction of High-Risk Livers Using Normothermic Machine Perfusion. LIVER TRANSPLANT.

[9] Nasralla D, Coussios CC, Mergental H (2018). A randomized trial of normothermic preservation in liver transplantation. NATURE.

[10] Dutkowski P, Linecker M, DeOliveira ML (2015). Challenges to Liver Transplantation and Strategies to Improve Outcomes. GASTROENTEROLOGY.

[11] Vodkin I, Kuo A (2017). Extended Criteria Donors in Liver Transplantation. CLIN LIVER DIS.

[12] Pagano D, Barbàra M, Seidita A (2020). Impact of Extended-Criteria Donor Liver Grafts on Benchmark Metrics of Clinical Outcome After Liver Transplantation: A Single Center Experience. TRANSPL P.

[13] Nemes B, Gaman G, Polak WG (2016). Extended-criteria donors in liver transplantation Part II: reviewing the impact of extended-criteria donors on the complications and outcomes of liver transplantation. Expert Rev Gastroenterol Hepatol.

[14] Mateo R, Cho Y, Singh G (2006). Risk Factors for Graft Survival After Liver Transplantation from Donation After Cardiac Death Donors: An Analysis of OPTN/UNOS Data. AM J TRANSPLANT.

[15] Skaro AI, Jay CL, Baker TB (2009). The impact of ischemic cholangiopathy in liver transplantation using donors after cardiac death: The untold story. SURGERY.

[16] Jay C, Ladner D, Wang E (2011). A comprehensive risk assessment of mortality following donation after cardiac death liver transplant - an analysis of the national registry. J HEPATOL.

[17] O'Neill S, Roebuck A, Khoo E (2014). A meta-analysis and meta-regression of outcomes including biliary complications in donation after cardiac death liver transplantation. TRANSPL INT.

[18] Jochmans I, Akhtar MZ, Nasralla D (2016). Past, Present, and Future of Dynamic Kidney and Liver Preservation and Resuscitation. AM J TRANSPLANT.

[19] Muller X, Marcon F, Sapisochin G (2018). Defining Benchmarks in Liver Transplantation. ANN SURG.

[20] Minor T, Efferz P, Fox M (2013). Controlled oxygenated rewarming of cold stored liver grafts by thermally graduated machine perfusion prior to reperfusion. AM J TRANSPLANT.

[21] Zhang Y, Zhang Y, Zhang M (2019). Hypothermic machine perfusion reduces the incidences of early allograft dysfunction and biliary complications and improves 1-year graft survival after human liver transplantation. MEDICINE.

[22] Guarrera JV, Henry SD, Samstein B (2010). Hypothermic Machine Preservation in Human Liver Transplantation: The First Clinical Series. AM J TRANSPLANT.

[23] van Rijn R, Karimian N, Matton APM (2017). Dual hypothermic oxygenated machine perfusion in liver transplants donated after circulatory death. BRIT J SURG.

[24] Werner MJM, Leeuwen OB, Jong IEM (2019). First report of successful transplantation of a pediatric donor liver graft after hypothermic machine perfusion. PEDIATR TRANSPLANT.

[25] Ishii D, Matsuno N, Gochi M (2020). Applicability of Hypothermic Oxygenate Machine Perfusion Preservation for Split-Liver Transplantation in a Porcine Model: An Experimental Study. ANN TRANSPL.

[26] Gillooly AR, Perry J, Martins PN (2019). First Report of siRNA Uptake (for RNA Interference) During *Ex vivo* Hypothermic and Normothermic Liver Machine Perfusion. TRANSPLANTATION.

[27] Selten J, Schlegel A, de Jonge J, Dutkowski P (2017). Hypo- and normothermic perfusion of the liver: Which way to go?. Best Practice & Research Clinical Gastroenterology.

[28] Schlegel A, Muller X, Dutkowski P (2018). Hypothermic Machine Preservation of the Liver: State of the Art. Current Transplantation Reports.

[29] Kron P, Schlegel A, Mancina L (2018). Hypothermic oxygenated perfusion (HOPE) for fatty liver grafts in rats and humans. J HEPATOL.

[30] Schlegel A, Kron P, De Oliveira ML (2016). Is single portal vein approach sufficient for hypothermic machine perfusion of DCD liver grafts?. J HEPATOL.

[31] van Rijn R, van Leeuwen OB, Matton A (2018). Hypothermic oxygenated machine perfusion reduces bile duct reperfusion injury after transplantation of donation after circulatory death livers. Liver Transpl.

[32] Ravaioli M, De Pace V, Angeletti A (2020). Hypothermic Oxygenated New Machine Perfusion System in Liver and Kidney Transplantation of Extended Criteria Donors:First Italian Clinical Trial. SCI REP-UK.

[33] Rayar M, Maillot B, Bergeat D (2018). A Preliminary Clinical Experience Using Hypothermic Oxygenated Machine Perfusion for Rapid Recovery of Octogenarian Liver Grafts. PROG TRANSPLANT.

[34] Lué A, Solanas E, Baptista P (2016). How important is donor age in liver transplantation?. WORLD J GASTROENTERO.

[35] Schlegel A, Muller X, Kalisvaart M (2019). Outcomes of DCD liver transplantation using organs treated by hypothermic oxygenated perfusion before implantation. J HEPATOL.

[36] De Carlis R, Lauterio A, Ferla F (2017). Hypothermic Machine Perfusion of Liver Grafts Can Safely Extend Cold Ischemia for Up to 20 Hours in Cases of Necessity. TRANSPLANTATION.

[37] Brüggenwirth IMA, van Leeuwen OB, de Vries Y (2020). Extended hypothermic oxygenated machine perfusion enables ex situ preservation of porcine livers for up to 24 hours. JHEP Reports.

[38] Bruinsma BG, Yeh H, Özer S (2014). Subnormothermic Machine Perfusion for*Ex vivo* Preservation and Recovery of the Human Liver for Transplantation. AM J TRANSPLANT.

[39] Kakizaki Y, Miyagi S, Shimizu K (2018). Effects of Subnormothermic Perfusion Before Transplantation for Liver Grafts from Donation After Cardiac Death: A Simplified Dripping Perfusion Method in Pigs. Transplant Proc.

[40] Olschewski P, Gaß P, Ariyakhagorn V (2010). The influence of storage temperature during machine perfusion on preservation quality of marginal donor livers. CRYOBIOLOGY.

[41] Morito N, Obara H, Matsuno N (2018). Oxygen consumption during hypothermic and subnormothermic machine perfusions of porcine liver grafts after cardiac death. J ARTIF ORGANS.

[42] Kanazawa H, Obara H, Yoshikawa R (2020). Impact of Machine Perfusion on Sinusoid Microcirculation of Liver Graft Donated After Cardiac Death. J SURG RES.

[43] Ciria R, Ayllon-Teran MD, González-Rubio S (2019). Rescue of Discarded Grafts for Liver Transplantation by *Ex vivo* Subnormothermic and Normothermic Oxygenated Machine Perfusion: First Experience in Spain. TRANSPL P.

[44] Shonaka T, Matsuno N, Obara H (2018). Application of Perfusate With Human-Derived Oxygen Carrier Solution Under Subnormothermic Machine Perfusion for Donation After Cardiac Death Liver Grafts in Pigs. TRANSPL P.

[45] Shonaka T, Matsuno N, Obara H (2019). Impact of human-derived hemoglobin based oxygen vesicles as a machine perfusion solution for liver donation after cardiac death in a pig model. PLOS ONE.

[46] Karimian N, Raigani S, Huang V (2019). Subnormothermic Machine Perfusion of Steatotic Livers Results in Increased Energy Charge at the Cost of Anti-Oxidant Capacity Compared to Normothermic Perfusion. Metabolites.

[47] Alva N, Panisello-Rosello A, Flores M (2018). Ubiquitin-proteasome system and oxidative stress in liver transplantation. World J Gastroenterol.

[48] Huang V, Karimian N, Detelich D (2020). Split-Liver Ex Situ Machine Perfusion: A Novel Technique for Studying Organ Preservation and Therapeutic Interventions. J CLIN MED.

[49] Obara H, Morito N, Matsuno N (2018). Optimum Perfusate Volume of Purified Subnormothermic Machine Perfusion for Porcine Liver Donated After Cardiac Death. TRANSPL P.

[50] Obara H, Morito N, Matsuno N (2020). Initial perfusate purification during subnormothermic machine perfusion for porcine liver donated after cardiac death. J ARTIF ORGANS.

[51] Tabka D, Bejaoui M, Javellaud J (2018). Angiotensin IV improves subnormothermic machine perfusion preservation of rat liver graft. BIOMED PHARMACOTHER.

[52] Hessheimer AJ, Fondevila C, García-Valdecasas JC (2012). Extracorporeal machine liver perfusion. CURR OPIN ORGAN TRAN.

[53] Vogel T, Brockmann JG, Coussios C, Friend PJ (2012). The role of normothermic extracorporeal perfusion in minimizing ischemia reperfusion injury. TRANSPLANT REV-ORLAN.

[54] Ravikumar R, Jassem W, Mergental H (2016). Liver Transplantation After *Ex vivo* Normothermic Machine Preservation: A Phase 1 (First-in-Man) Clinical Trial. AM J TRANSPLANT.

[55] Zhang Z, Ju W, Tang Y (2020). First Preliminary Experience with Preservation of Liver Grafts from Extended-Criteria Donors by Normothermic Machine Perfusion in Asia. ANN TRANSPL.

[56] Ceresa C, Nasralla D, Watson C (2019). Transient Cold Storage Prior to Normothermic Liver Perfusion May Facilitate Adoption of a Novel Technology. Liver Transpl.

[57] Stephenson BTF, Bonney GK, Laing RW (2018). Proof of concept: liver splitting during normothermic machine perfusion. Journal of Surgical Case Reports. 2018.

[58] Laing RW, Bhogal RH, Wallace L (2017). The Use of an Acellular Oxygen Carrier in a Human Liver Model of Normothermic Machine Perfusion. TRANSPLANTATION.

[59] Liu Q, Hassan A, Pezzati D (2020). Ex Situ Liver Machine Perfusion: The Impact of Fresh Frozen Plasma. Liver Transpl.

[60] Selzner M, Goldaracena N, Echeverri J (2016). Normothermic *ex vivo* liver perfusion using steen solution as perfusate for human liver transplantation: First North American results. Liver Transpl.

[61] Thijssen MF, Brüggenwirth IMA, Gillooly A (2019). Gene Silencing With siRNA (RNA Interference): A New Therapeutic Option During *Ex vivo* Machine Liver Perfusion Preservation. LIVER TRANSPLANT.

[62] Todo S, Yamashita K, Goto R (2016). A pilot study of operational tolerance with a regulatory T-cell-based cell therapy in living donor liver transplantation. HEPATOLOGY.

[63] Rigo F, De Stefano N, Navarro-Tableros V (2018). Extracellular Vesicles from Human Liver Stem Cells Reduce Injury in an *Ex vivo* Normothermic Hypoxic Rat Liver Perfusion Model. TRANSPLANTATION.

[64] Hara Y, Akamatsu Y, Maida K (2013). A new liver graft preparation method for uncontrolled non-heart-beating donors, combining short oxygenated warm perfusion and prostaglandin E1. J SURG RES.

[65] Maida K, Akamatsu Y, Hara Y (2016). Short Oxygenated Warm Perfusion With Prostaglandin E1 Administration Before Cold Preservation as a Novel Resuscitation Method for Liver Grafts From Donors After Cardiac Death in a Rat *In vivo* Model. TRANSPLANTATION.

[66] Goldaracena N, Spetzler VN, Echeverri J (2017). Inducing Hepatitis C Virus Resistance After Pig Liver Transplantation-A Proof of Concept of Liver Graft Modification Using Warm*Ex vivo* Perfusion. AM J TRANSPLANT.

[67] Boteon YL, Boteon APCS, Attard J (2018). Ex situ machine perfusion as a tool to recondition steatotic donor livers: Troublesome features of fatty livers and the role of defatting therapies. A systematic review. AM J TRANSPLANT.

[68] Nagrath D, Xu H, Tanimura Y (2009). Metabolic preconditioning of donor organs: Defatting fatty livers by normothermic perfusion *ex vivo*. METAB ENG.

[69] Boteon YL, Attard J, Boteon A (2019). Manipulation of Lipid Metabolism During Normothermic Machine Perfusion: Effect of Defatting Therapies on Donor Liver Functional Recovery. Liver Transpl.

[70] Beal EW, Kim J, Reader BF (2019). [D-Ala2, D-Leu5] Enkephalin Improves Liver Preservation During Normothermic *Ex vivo* Perfusion. J SURG RES.

[71] Hoyer DP, Mathé Z, Gallinat A (2016). Controlled Oxygenated Rewarming of Cold Stored Livers Prior to Transplantation. TRANSPLANTATION.

[72] Hoyer DP, Benkö T, Manka P (2020). Long-term Outcomes After Controlled Oxygenated Rewarming of Human Livers Before Transplantation. Transplantation Direct.

[73] Czigany Z, Tacke F, Lurje G (2019). Evolving Trends in Machine Liver Perfusion: Comments on Clinical End Points and Selection Criteria. GASTROENTEROLOGY.

[74] Boteon YL, Afford SC (2019). Machine perfusion of the liver: Which is the best technique to mitigate ischaemia-reperfusion injury?. World J Transplant.

[75] Muller X, Schlegel A, Kron P (2019). Novel Real-time Prediction of Liver Graft Function During Hypothermic Oxygenated Machine Perfusion Before Liver Transplantation. ANN SURG.

[76] Karangwa SA, Dutkowski P, Fontes P (2016). Machine Perfusion of Donor Livers for Transplantation: A Proposal for Standardized Nomenclature and Reporting Guidelines. AM J TRANSPLANT.

[77] van Leeuwen OB, de Vries Y, Fujiyoshi M (2019). Transplantation of High-risk Donor Livers After Ex Situ Resuscitation and Assessment Using Combined Hypo- and Normothermic Machine Perfusion. ANN SURG.

[78] Boteon YL, Laing RW, Schlegel A (2019). The impact on the bioenergetic status and oxidative-mediated tissue injury of a combined protocol of hypothermic and normothermic machine perfusion using an acellular haemoglobin-based oxygen carrier: The cold-to-warm machine perfusion of the liver. PLOS ONE.

[79] de Vries Y, Berendsen TA, Fujiyoshi M (2019). Transplantation of high-risk donor livers after resuscitation and viability assessment using a combined protocol of oxygenated hypothermic, rewarming and normothermic machine perfusion: study protocol for a prospective, single-arm study (DHOPE-COR-NMP trial). BMJ OPEN.

[80] de Vries RJ, Tessier SN, Banik PD (2019). Supercooling extends preservation time of human livers. NAT BIOTECHNOL.

[81] Eshmuminov D, Becker D, Bautista Borrego L (2020). An integrated perfusion machine preserves injured human livers for 1 week. NAT BIOTECHNOL.

